# Systemic immune dysregulation and neutrophil activation define prognostic inflammatory signatures in drug-resistant epilepsy

**DOI:** 10.1172/jci.insight.200419

**Published:** 2026-04-14

**Authors:** Coraly Simoës Da Gama, Aurélie Hanin, Gwen Goudard, Véronique Masson, Aurore Besnard, Karim Dorgham, Guy Gorochov, Guillaume Dorothée, Valerio Frazzini, Vincent Navarro, Mélanie Morin-Brureau

**Affiliations:** 1 Sorbonne Université, Inserm, Centre de Recherche Saint-Antoine (CRSA), Hôpital Saint-Antoine, Paris, France.; 2 Sorbonne Université, Paris Brain Institute, ICM, INSERM, CNRS, AP-HP, Pitié-Salpêtrière Hospital, Paris, France.; 3 AP-HP, Sorbonne Université, Pitié-Salpêtrière - Charles Foix University Hospital, Department of Metabolic Biochemistry, DMU BioGeMH, Paris, France.; 4 Université Paris Cité, Faculté de Pharmacie, Paris, France.; 5 AP-HP, Epilepsy Unit, Pitié-Salpêtrière Hospital, Paris, France.; 6 Sorbonne Université, Inserm, Centre d’Immunologie et des Maladies Infectieuses, AP-HP, Département d’Immunologie, Hôpital de la Pitié-Salpêtrière, Paris, France.; 7 Center of Reference for Rare Epilepsies, ERN EPICARE, AP-HP, Pitié-Salpêtrière Hospital, Paris, France.

**Keywords:** Immunology, Inflammation, Neuroscience, Epilepsy, Neutrophils

## Abstract

Systemic inflammation is now recognized as a key contributor to epilepsy pathophysiology, yet the role of innate immune cells, particularly neutrophils, remains poorly defined in epilepsy. Preclinical studies in rodent models have implicated neutrophils in seizure activity, but their phenotype in human epilepsy has not been thoroughly investigated. In this study, we aimed to characterize systemic inflammatory profiles and neutrophil-associated immune signatures in the blood of patients with drug-resistant epilepsy compared with healthy controls. We identified a systemic low-grade inflammatory profile in patients characterized by elevated neutrophil-to-lymphocyte ratio, C-reactive protein, proinflammatory cytokines (IL-6, CXCL8/IL-8, TNF-α), and activated neutrophils (CXCR4^+^CD62L^lo^). Neutrophil phenotyping revealed two distinct immune profiles. Patients with longer disease duration exhibited a more immature systemic signature characterized by immature neutrophils (CD15^+^CD10^–^), resting neutrophils (CXCR4^+^CD62L^+^), and elevated IL-6 levels. In contrast, patients with higher seizure frequency displayed a more inflammatory profile, marked by increased IL-12 and activated (CXCR4^+^CD62L^lo^) and hyperactivated (CXCR4^hi^CD62L^lo^) neutrophil subsets. Moreover, elevated presurgical levels of inflammatory profile TNF-α, IL-6, and hyperactivated CXCR4^hi^CD62L^lo^ neutrophils were associated with seizure recurrence 1 year after surgery. This pioneering study highlights the heterogeneity of peripheral immune responses in drug-resistant epilepsy and identifies neutrophil-related signatures as promising prognostic biomarkers in this context.

## Abstract 2

In drug-resistant epilepsy, systemic inflammation linked to overactive neutrophils may serve as a biomarker of disease severity and risk of seizure recurrence after surgery.

## Graphical abstract

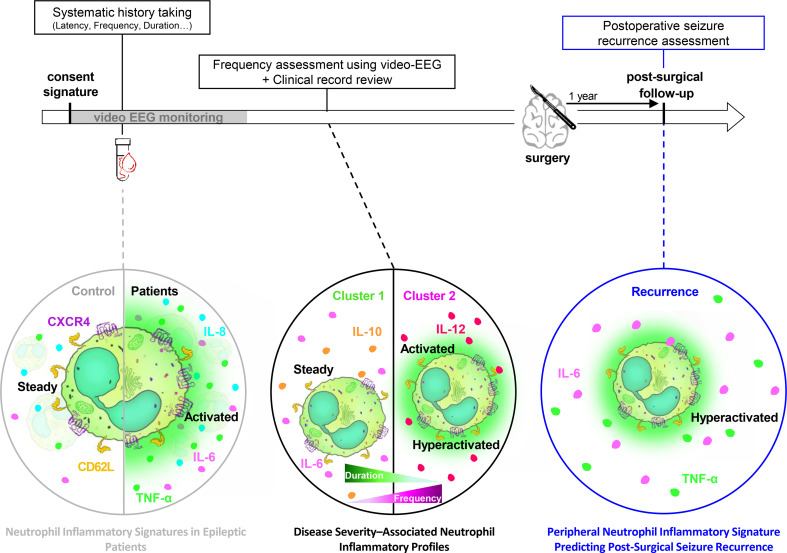

## Introduction

Both systemic and CNS inflammation have been implicated in epilepsy pathophysiology, although the nature and magnitude of immune activation vary across etiologies, including autoimmune and paraneoplastic encephalitis as well as traumatic brain injury (1, 2). A compromised blood-brain barrier (BBB) facilitates crosstalk between peripheral and central systems. Systemic inflammation contributes to increased seizure susceptibility in rodent models (3, 4) and patients (4, 5), activating peripheral immune cells to release proinflammatory cytokines like IL-1β or TNF-α (6, 7), thereby enhancing neuronal hyperexcitability and promoting seizure recurrence (8, 9).

Investigations on specific subpopulations of peripheral immune cells found an increase in proinflammatory regulatory B cells and a decrease in Treg activity in the blood of patients with focal epilepsy (10). Similarly, a shift in CD4^+^ T cells toward a proinflammatory Th1 phenotype has been observed in patients with drug-resistant epilepsy (DRE) (11) and has been associated with changes in microglial dynamics in rodent models (12). Altered monocyte profiles have also been identified in patients 1 day after seizures (13), and proinflammatory monocytes were found in patients with DRE (14).

Neutrophils are increasingly recognized as heterogeneous immune cells whose phenotypic diversity is shaped by maturation state, proliferative capacity, and short lifespan (15). They can rapidly adapt to environmental stimuli by altering their transcriptome (16, 17), epigenetic landscape (18), cellular functions (17), and surface molecule expression (19). This plasticity contributes to the emergence of distinct neutrophil subtypes, which play critical roles in the pathophysiology and prognosis of various diseases, including Alzheimer disease and cerebral ischemia (20, 21).

In the context of epilepsy, evidence from both animal models and human studies suggests that neutrophils may contribute to disease mechanisms. In rodent models of temporal lobe epilepsy (TLE), neutrophil adhesion to cerebral vessels has been shown to reduce blood flow during the postictal phase (22). Neutrophil infiltration into the brain has been observed after seizures (23), where it has been associated with neuronal death (24), while neutrophil depletion reduces seizure severity (25). In patients with epilepsy, increased blood neutrophil counts have been correlated with seizure severity (26, 27), seizure recurrence (27), and posttraumatic epilepsy (28). Moreover, elevated levels of CXCL8/IL-8, a key chemokine involved in neutrophil activation and recruitment, have been detected in the blood (29, 30), cerebrospinal fluid (29), and brain tissue (31) of patients with hippocampal sclerosis TLE.

Further supporting a role for neutrophils in seizure-related inflammation, patients with anti-NMDAR encephalitis exhibit increased production of neutrophil extracellular traps (32), and altered granulocyte phenotypes have been observed after seizures, including reduced MHC-II expression and increased Fcγ receptor levels (13).

Despite this growing body of evidence, the phenotypic characteristics of circulating peripheral neutrophils in epilepsy remain largely unexplored. Characterizing these phenotypes could offer new insights into the immune mechanisms contributing to seizure burden and epilepsy progression.

In this study, we aimed to characterize the inflammatory environment and the phenotypic subsets of neutrophils in patients with DRE, mainly temporal DRE, which is the most common DRE in adults. We further investigated whether clinical features such as the timing of the last seizure, seizure frequency, or disease duration could influence the neutrophil-related inflammation burden. Finally, we explored whether specific inflammatory signatures, particularly those involving neutrophil subsets, could be associated with, or predictive of, seizure recurrence after epilepsy surgery.

## Results

### Population of patients with DRE.

Serum samples were collected from 67 patients with DRE (median age 35 years [IQR 28–47]) and 35 age-matched healthy controls. Clinical timing variables were defined using standardized criteria. Disease duration corresponded to the interval between first seizure and blood sampling. Seizure frequency and latency were estimated from patient reports, and seizure frequency was corroborated by video electroencephalogram (EEG) when available. Thirty-two patients had seizures within 48 hours before sampling (“post seizure,” median 0.66 hours [0.33–1]), and 31 patients had samples collected more than 48 hours after the last seizure (“baseline,” median 7 [4–10]). Most patients had TLE (*n* = 54, 81%). Regarding seizure frequency, patients experienced multiple seizures daily (*n* = 15), weekly (*n* = 32), monthly (*n* = 15), and annually (*n* = 4). Median disease duration was 17 years [IQR 8–28]. Among the 67 patients, 49 (73%) underwent epilepsy surgery, with neuropathology revealing hippocampal sclerosis (*n* = 25, 51%); tumors (*n* = 13, 26%); and other lesions such as dysplasia, gliosis, or cavernoma (*n* = 11, 22%). Seizures were mostly focal with impaired awareness (*n* = 40, 60%), and about half of the patients experienced multi-weekly seizures (*n* = 32, 48%). Information regarding post-surgery outcomes was available for 48 patients and revealed seizure recurrence for 15 of them (47%) at 1 year after surgery. All clinical features are detailed in Table 1. Information regarding antiseizure medications was collected for all patients. Patients receiving systemic glucocorticoids or immunotherapy at the time of blood sampling were excluded to avoid confounding effects on leukocyte activation. Among the 110 patients, 17 different antiseizure medication molecules were used, with most patients receiving polytherapy (> 100 distinct combinations). Comparison between patients receiving antiseizure medications with reported antiinflammatory effects (e.g., valproate, levetiracetam) and those not receiving them revealed no significant differences in neutrophil activation or inflammatory markers. Similarly, grouping antiseizure medications by mechanism of action did not reveal significant associations (data not shown).

### Peripheral immune dysregulation reflected by altered blood cell counts and neutrophil activation states.

**Figure 1:**
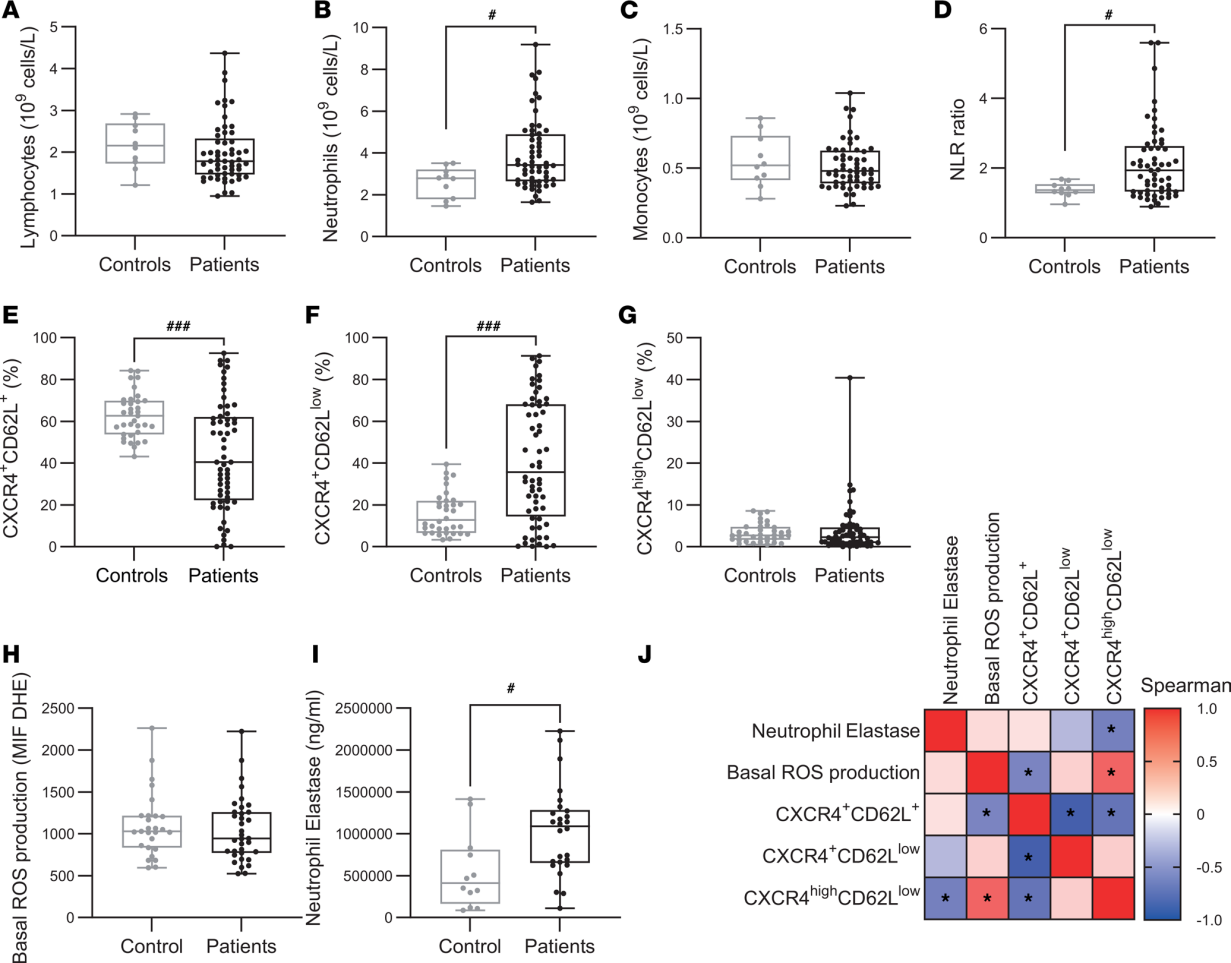
Peripheral immune profiling and neutrophil activation states in healthy controls and patients with epilepsy. (**A**–**D**) Quantification of lymphocytes, neutrophils, monocytes, and neutrophil-to-lymphocyte (NLR) ratio in whole blood from healthy controls (gray, *n* = 10) and patients with epilepsy (black, *n* = 57). (**E**–**G**) Percentage of CXCR4^+^CD62^+^ (**E**), CXCR4^+^CD62L^lo^ (**F**), and CXCR4^hi^CD62L^lo^ (**G**) neutrophils in control (gray, *n* = 42) and patients with epilepsy (black, *n* = 59). (**H**) Basal ROS production capacity in neutrophils from controls (gray, *n* = 27) and patients with epilepsy (black, *n* = 35). (**I**) Serum neutrophil elastase concentration in controls (gray, *n* = 12) and patients with epilepsy (black, *n* = 26). (**J**) Correlation table showing Spearman’s correlation coefficients between basal ROS production, serum neutrophil elastase concentration, and the 3 neutrophil activation states: CXCR4^+^CD62^+^, CXCR4^+^CD62L^lo^, and CXCR4^hi^CD62L^lo^. Normality was assessed using the Shapiro-Wilk test; comparisons were performed using 2-tailed Student’s *t* test or Mann-Whitney U test as appropriate. #*P* < 0.05, ###*P* < 0.001. Correlations were analyzed using Spearman’s rank correlation test.

Compared with healthy controls, patients with DRE exhibited a significant increase in neutrophil/lymphocyte ratios (1.365 vs. 1,940, *P* = 0.02), and neutrophil counts (2.78 × 10^9^/L vs. 3.42 × 10^9^/L, *P* = 0.013; normal range: 2.5–7 × 10^9^/L), with no significant change in lymphocyte and monocyte counts (Figure 1, A–D).

To further characterize neutrophil involvement, we assessed their activation state using flow cytometry based on CXCR4 and CD62L expression. This approach allowed us to distinguish 3 activation subsets, steady state (CXCR4^+^CD62L^+^), activated (CXCR4^+^CD62L^lo^), and hyperactivated (CXCR4^hi^CD62L^lo^), according to our gating strategy (33). Briefly, after removing doublets in the neutrophil population, CXCR4^hi^ and CD62L^lo^ were determined in the control group using a mean intensity fluorescence (MIF) histogram and then applied in all patients (Supplemental Figure 1; supplemental material available online with this article; https://doi.org/10.1172/jci.insight.200419DS1). Patients with DRE showed a distinct activation pattern compared with healthy controls, with a reduced frequency of steady-state CXCR4^+^CD62L^+^ neutrophils (62.7% vs. 40.4%; *P* < 0.0001) and a higher percentage of activated CXCR4^+^CD62L^lo^ neutrophils (12.8% vs. 35.7%; *P* = 0.0001), while the proportion of hyperactivated CXCR4^hi^CD62L^lo^ neutrophils (2.75% vs. 2.18%) remained unchanged (Figure 1, E–G).

To further investigate basal neutrophil activation in patients with DRE, we assessed their degranulation capacity and ROS production. No differences were observed in basal ROS output between patients and healthy controls (Figure 1H). In contrast, neutrophil elastase levels were significantly elevated in the serum of patients with DRE compared with controls (Figure 1I). We then examined correlations between these functional markers and neutrophil activation phenotypes. Interestingly, the proportion of CXCR4^hi^CD62L^lo^ neutrophils correlated positively with ROS levels but negatively with serum neutrophil elastase levels (Figure 1J). Altogether, these data support a higher basal activation of neutrophils in patients with DRE compared with healthy controls with a higher degranulation capacity.

### Chronic peripheral low-grade inflammation in patients with DRE.

**Figure 2:**
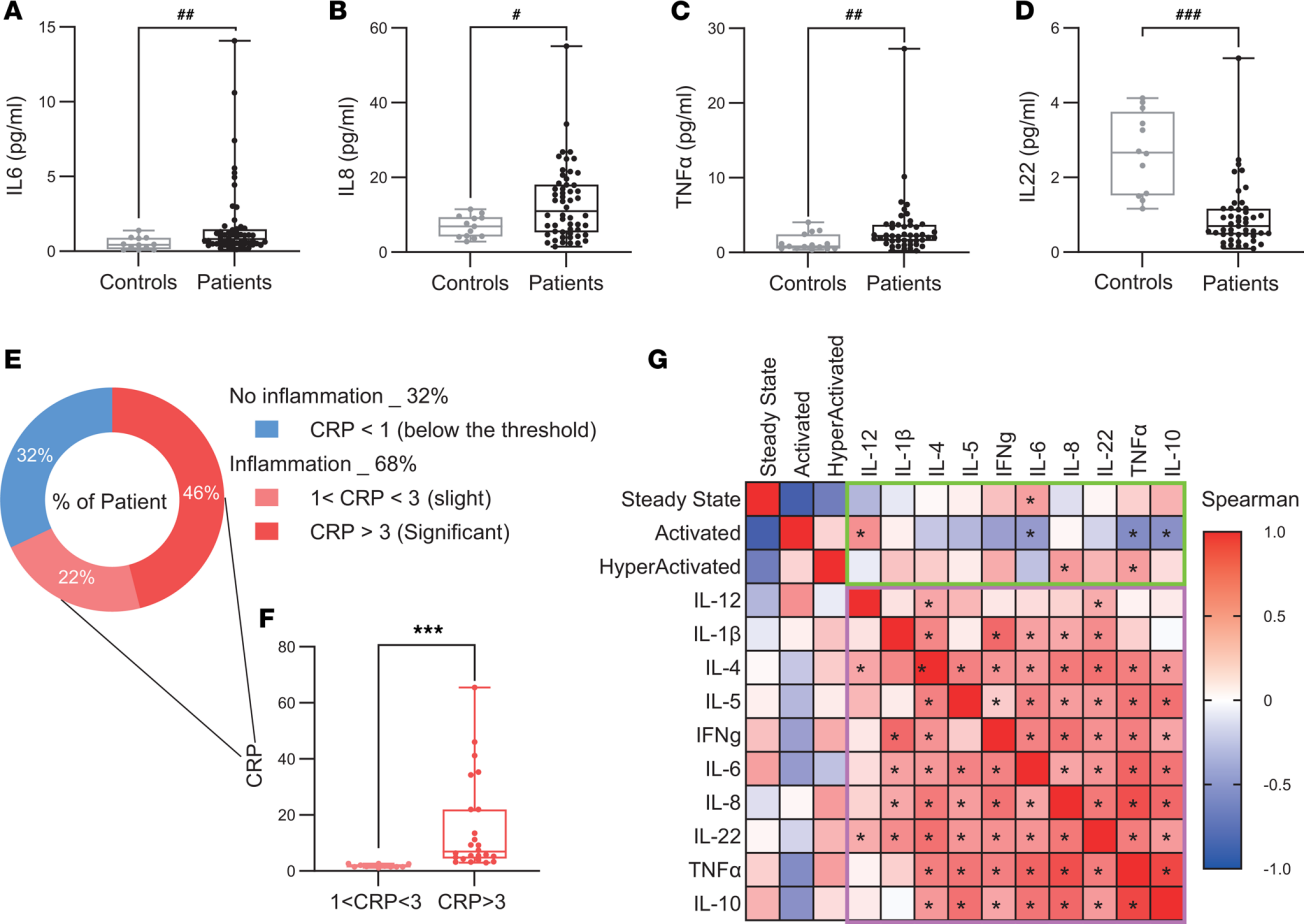
Chronic peripheral low-grade inflammation in patients with epilepsy. (**A**–**D**) Graphics displaying cytokine concentrations in sera of control individuals (gray, *n* = 12) and patients with epilepsy (black, *n* = 48) and expressed in pg/mL. (**E**) Distribution of inflammation assessed by CRP in patients with epilepsy: normal inflammation (CRP < 1, blue), slight inflammation (1 < CRP < 3, light red), significant (CRP > 3, red) and (**F**) histogram showing the distribution of CRP levels in patients (*n* = 36), categorized as moderate (1 < CRP ≤ 3 mg/L) and high (CRP > 3 mg/L). (**G**) Heatmap showing Spearman’s correlation coefficients (*r*) between serum cytokine levels in patients with epilepsy (pink square outline) and neutrophil activation states in blood (green square outline). Color intensity reflects the strength and direction of correlation, with red indicating positive correlations and blue indicating negative correlations. Normality was assessed using the Shapiro-Wilk test; comparisons were performed using 2-tailed Student’s *t* test or Mann-Whitney U test as appropriate. #*P* < 0.05, ###*P* < 0.001. Statistical comparisons between patients and controls are denoted by ^#^, whereas comparisons among different patient groups are denoted by *.

To further investigate the inflammatory environment, we measured the levels of 10 cytokines in nonepileptic and epileptic sera (Figure 2, A–D, and Supplemental Figure 2). Serum analysis revealed elevated levels of proinflammatory cytokines in patients with DRE compared with healthy controls: IL-6 (0.83 vs. 0.41 pg/mL; *P* = 0.005), CXCL8/IL-8 (10.96 vs. 6.92 pg/mL; *P* = 0.03, I, and TNF-α (2.23 vs. 0.82 pg/mL; *P* = 0.006); antiinflammatory cytokine IL-22 levels were reduced (0.70 vs. 2.66 pg/mL; *P* < 0.0001) (Figure 2, A–D). Cluster analysis revealed significant positive correlations among most cytokines (Figure 2G, pink square outline).

Given the proinflammatory profile observed, we next assessed C-reactive protein (CRP) levels as a general marker of systemic inflammation. Based on established thresholds (CRP < 1 mg/L: no inflammation; 1–3 mg/L: slight inflammation; > 3 mg/L: significant inflammation) (34, 35), we found that 22% of patients with DRE presented slightly elevated CRP (1.685 mg/mL), and 46% had CRP levels above 3 mg/L (6.9 mg/mL) (Figure 2, E and F).

To investigate a potential association between inflammatory cytokines and neutrophil phenotypes, we performed correlation analyses. We observed that steady-state CXCR4^+^CD62L^+^ neutrophils were positively correlated with IL-6 levels (*r* = 0.437, *P* = 0.001). In contrast, activated CXCR4+CD62L^lo^ neutrophils were negatively correlated with IL-6 (r = –0.472, P = 0.0004), TNF-α (r = –0.405, P = 0.008), and IL-10 (r = –0.358, P = 0.02) but positively correlated with IL-12 (r = 0.273, P = 0.05) (Figure 2G, green square outline). CXCR4^+^CD62L^lo^ neutrophils were negatively correlated with IL-6 (*r* = –0.472, *P* = 0.0004), TNF-α (*r* = –0.405, *P* = 0.008), and IL-10 (*r* = –0.358, *P* = 0.02) but positively correlated with IL-12 (*r* = 0.273, *P* = 0.05). Finally, hyperactivated CXCR4^hi^CD62L^lo^ neutrophils were positively correlated with IL-8 and TNF-α (IL-8_ *r* = 0.349, *P* = 0.01; TNF-α_ *r* = 0.347, *P* = 0.04).

Taken together, these findings indicate a peripheral low-grade chronic inflammation characterized by elevated levels of proinflammatory cytokines (CXCL8/IL-8, IL-6, TNF-α) and CRP and associated with an altered neutrophil activation in patients with DRE.

### Peripheral low-grade chronic inflammation is independent of a recent seizure.

Given that epileptic seizures are associated with increased inflammatory processes (7), we evaluated whether this low-grade inflammation was related to a recent seizure. When considering the delay since the last seizure as a continuous variable, no significant correlations were observed between seizure latency and neutrophil phenotypes or circulating cytokine levels (Table 2). We stratified our patients with DRE into 2 groups: patients who experienced a seizure within 48 hours before blood collection (< 48 hours, *n* = 32) and those who had been seizure-free for more than 48 hours (> 48 hours, *n* = 31). This 48-hour window was chosen based on previous studies showing that inflammatory cytokines, such as IL-6, return to baseline within approximately 48 hours (31, 36–38). Moreover, neutrophils exhibit rapid turnover in the circulation, with a residence time typically of several hours to less than 1–2 days, particularly under inflammatory conditions (39, 40). Patients were classified as baseline or “post seizure,” with samples collected a median of 7 and 0.5 days, respectively, after the last seizure.

**Figure 3:**
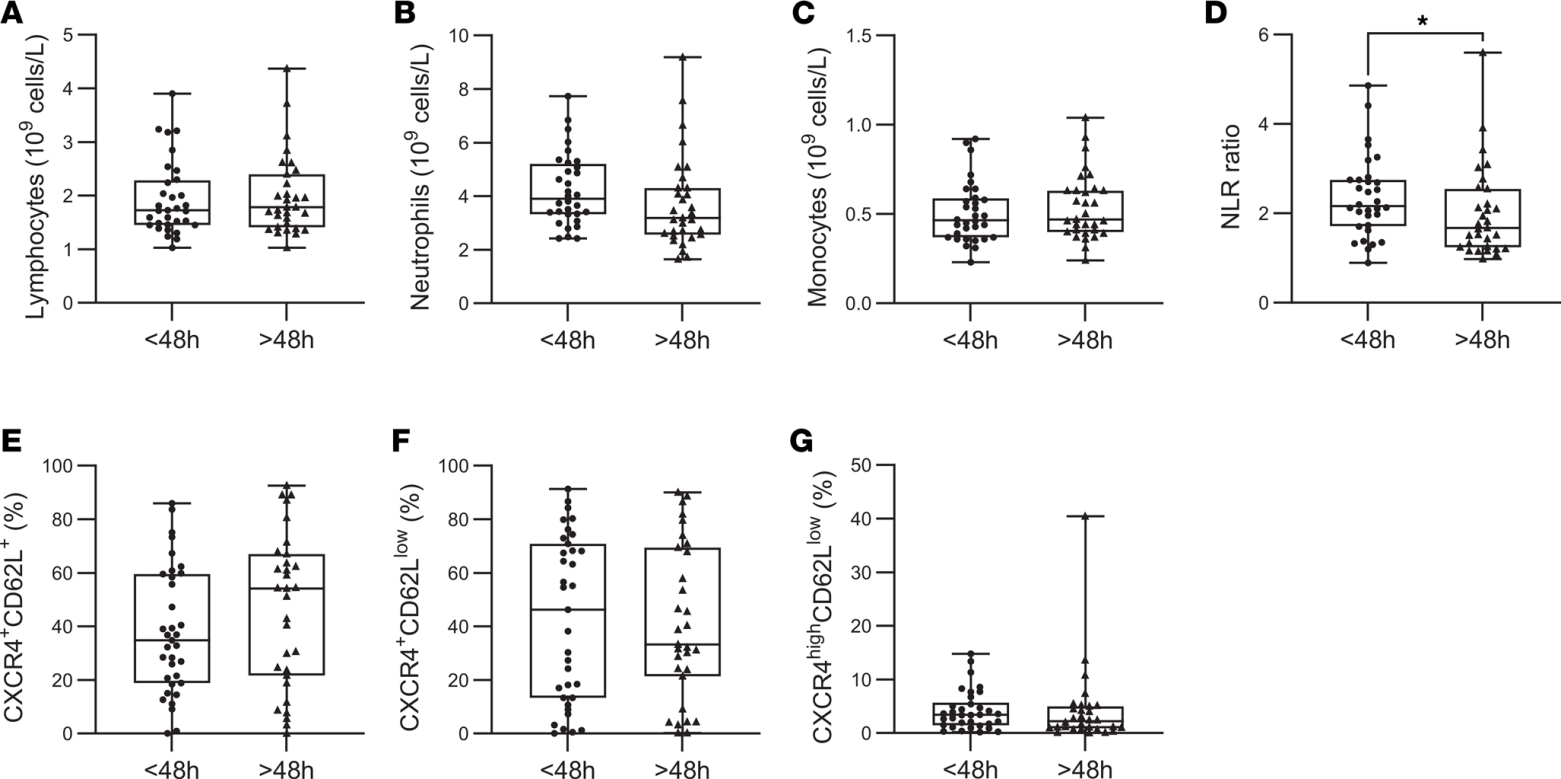
Neutrophil activation is independent of seizure latency. (**A**–**D**) Measurement of lymphocyte (**A**), neutrophil (**B**), and monocyte (**C**) counts, as well as the neutrophil-to-lymphocyte ratio (NLR) (**D**), in the whole blood of patients with epilepsy with a seizure within the last 48 hours (< 48 hours, *n* = 32) and those seizure-free for more than 48 hours (> 48 hours, *n* = 31). (**E**–**G**) Graphical representation of the distribution of CXCR4^+^CD62L^+^ (**E**), CXCR4^+^CD62L^lo^ (**F**), and CXCR4^hi^CD62L^lo^ (**G**) neutrophils in patients with epilepsy with a seizure within the last 48 hours (< 48 hours, *n* = 32) and those seizure-free for more than 48 hours (> 48 hours, *n* = 31). Normality was assessed using the Shapiro-Wilk test; comparisons were performed using 2-tailed Student’s *t* test or Mann-Whitney U test as appropriate. *P < 0.05.

**Figure 4:**
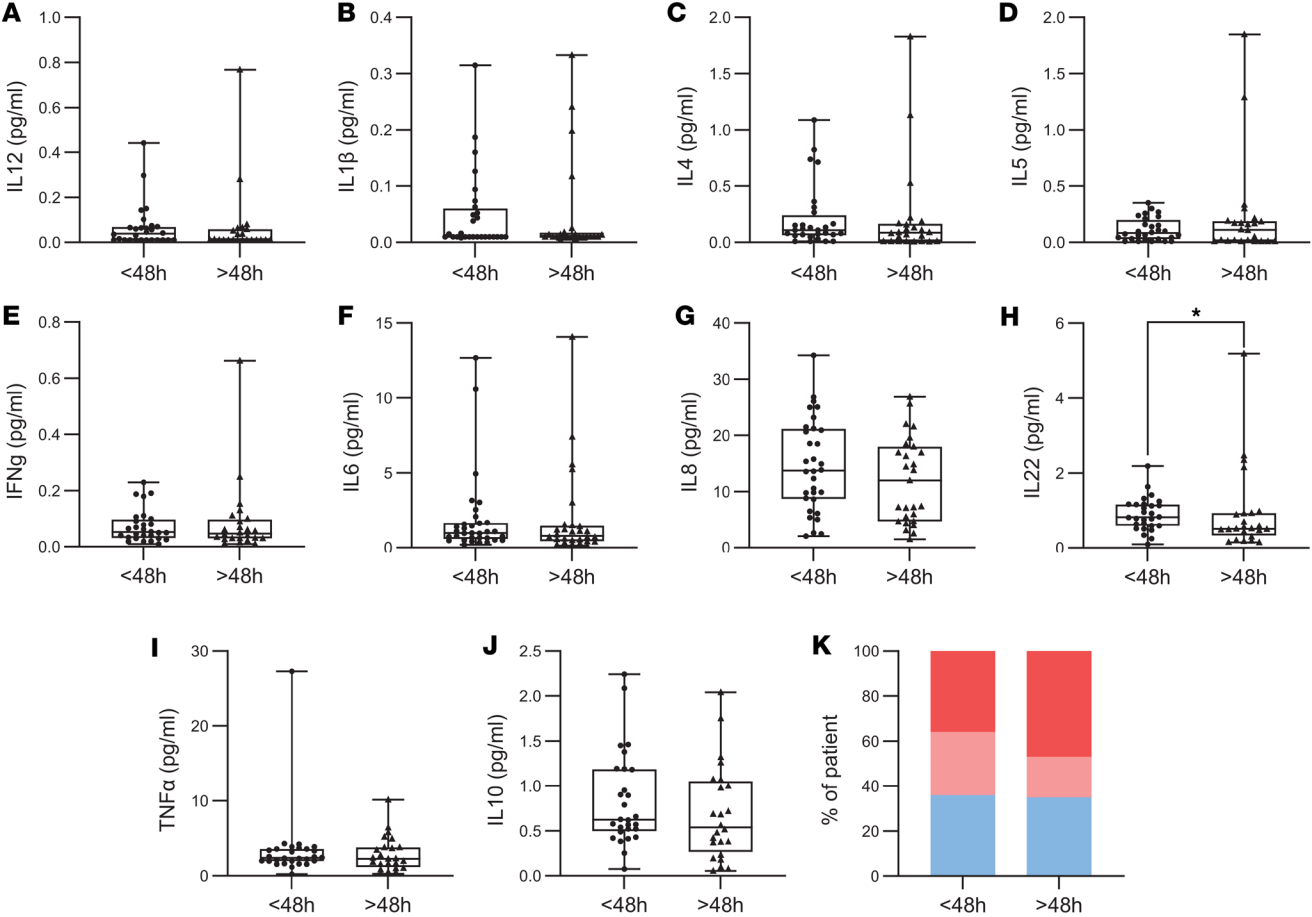
Chronic peripheral low-grade inflammation independent of seizure latency. (**A**–**J**) Graphics displaying cytokine concentrations between patients with epilepsy with a seizure within the last 48 hours (< 48 hours, *n* = 27) and those seizure-free for more than 48 hours (> 48 hours, *n* = 28). (**K**) Distribution of inflammation assessed by CRP in patients with epilepsy with a seizure within the last 48 hours and those seizure-free for more than 48 hours. No inflammation (CRP < 1, blue), slight inflammation (1 < CRP < 3, light red), significant (CRP > 3, red). Normality was assessed using the Shapiro-Wilk test; comparisons were performed using 2-tailed Student’s t test or Mann-Whitney U test as appropriate. For distribution comparisons, the χ^2^ test was used. *P < 0.05.

When comparing these 2 subgroups, no significant differences were found in the absolute counts of lymphocytes, neutrophils, or monocytes. However, the neutrophil/lymphocyte ratio was significantly higher in patients who had a seizure within the last 48 hours (2.26 vs. 1.67; *P* = 0.03) (Figure 3, A–D). Neutrophil counts were not elevated, and analysis of their activation states showed no significant differences between patients with recent seizures and those without across the 3 defined subpopulations (Figure 3, E–G). Among the cytokines analyzed, IL-22 was the only one found to be significantly elevated in patients who had experienced a seizure within the previous 48 hours, relative to those who had been seizure-free for more than 48 hours (0.8 vs. 0.5 pg/mL; *P* = 0.05) (Figure 4, A–J). Finally, we observed no significant difference in the CRP levels between these 2 subgroups of patients (Figure 4K). These findings suggest that although recent seizure activity may acutely influence systemic immune responses, the chronic low-grade inflammation observed in patients with DRE appears to persist independently of seizure timing.

### Two distinct inflammatory phenotypes identified in patients with DRE.

The clinical phenotype of patients with DRE is heterogeneous, primarily due to the diversity of clinical parameters in our cohort. We investigated the impact of the clinical parameters of lesion localization (i.e., temporal vs. others) and the presence of hippocampal sclerosis or other lesions, including tumors, on systemic inflammation. Despite a significant increase in lymphocyte counts and variability in CRP levels, these alterations did not seem to affect cytokine profiles or neutrophil phenotypic distribution (Supplemental Figures 3 and 4).

**Figure 5:**
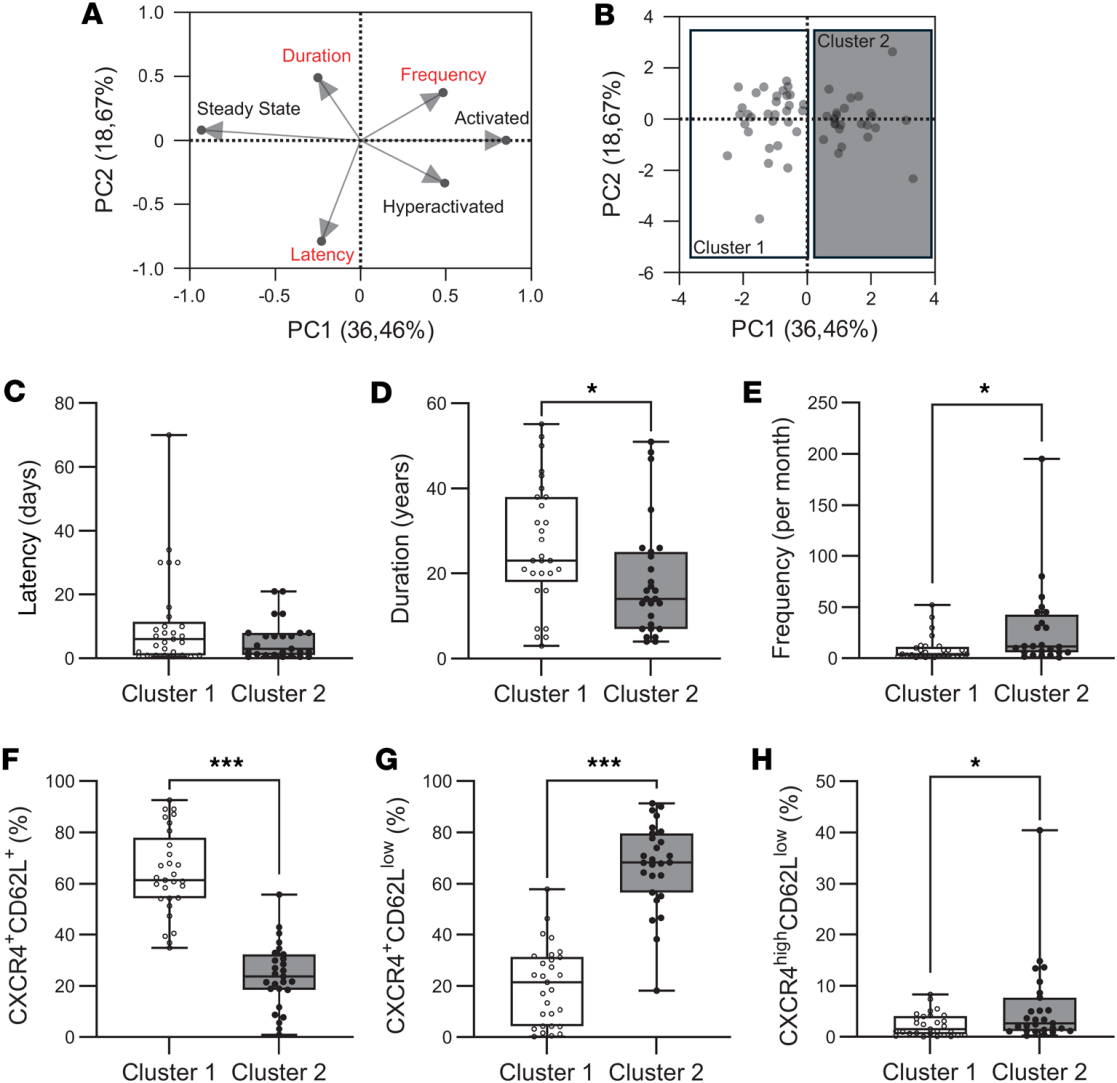
Patient stratification according to seizure frequency, duration of epilepsy, and neutrophil activation. (**A-B**) The PCA of 3 neutrophil phenotypes and 3 clinical variables for patients with epilepsy. Variable contribution in building the PCA is represented by the length of each arrow. Variable correlation plot illustrating the relationship between clinical data and activation state data. Graph depicting the distribution of individuals following PCA and the clustering of patients into 2 groups (*n* = 55). (**C**–**E**) Graphics displaying clinical data: latency (**C**), duration (**D**), and seizure frequency per month (**E**) among the 2 clusters (*n* = 29, cluster 1; *n* = 26, cluster 2). (**F**–**H**) Percentage of CXCR4^+^CD62^+^ (**F**), CXCR4^+^CD62L^lo^ (**G**), and CXCR4^hi^CD62L^lo^ (**H**) in each patients’ clusters (*n* = 29, cluster 1; *n* = 26, cluster 2). Normality was assessed using the Shapiro-Wilk test; comparisons were performed using 2-tailed Student’s t test or Mann-Whitney U test as appropriate. *P < 0.05, ***P < 0.001.

Then, we investigated the impact of monthly seizure frequency, disease duration, and latency of the most recent seizures on systemic inflammation. We performed a principal component analysis (PCA) integrating neutrophil activation states (steady, activated, hyperactivated) with 3 clinical parameters: seizure latency, epilepsy duration, and seizure frequency per month. This multivariate analysis revealed 2 distinct patient clusters (Figure 5, A and B).

An initial comparison of clinical characteristics between the 2 clusters revealed no significant difference in seizure latency, with both groups exhibiting a median of around 5 days (Figure 5C). Conversely, patients in cluster 1 had a significantly longer disease duration (23 vs. 14 years, *P* = 0.02), whereas those in cluster 2 experienced a substantially higher monthly seizure frequency (11.6 vs. 4 seizures/month, *P* = 0.01) (Figure 5, D and E). Regarding neutrophil activation, patients in cluster 1 exhibited a significantly higher proportion of steady-state neutrophils (CXCR4^+^CD62L^+^) compared with those in cluster 2 (61.3% vs. 23.7%, *P* < 0.0001) (Figure 5F). In contrast, cluster 2 showed increased proportions of both activated (CXCR4^+^CD62L^lo^) and hyperactivated (CXCR4^hi^CD62L^lo^) neutrophils (68.3% vs. 21.4%, *P* < 0.0001 and 2.6% vs. 1.5%, *P* = 0.03, respectively) (Figure 5, G and H).

**Figure 6:**
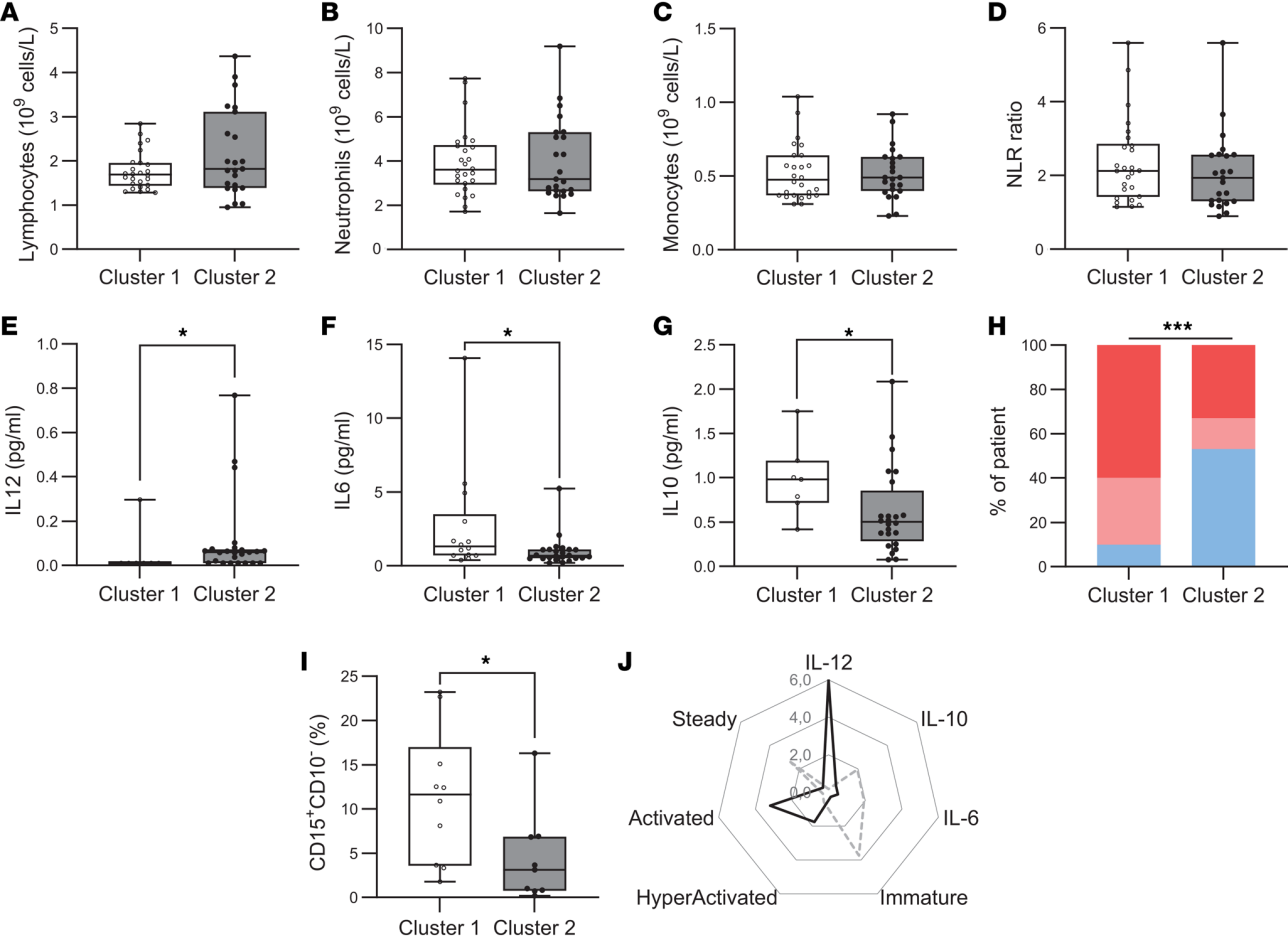
Characterization of peripheral inflammation in patients’ clusters. (**A**–**D**) Histogram representing lymphocyte (**A**), neutrophil (**B**), and monocyte (**C**) counts, as well as the neutrophil-to-lymphocyte ratio (NLR) (**D**), in the whole blood of patients with epilepsy of each cluster (*n* = 29, cluster 1; *n* = 26 cluster2). (**E**–**G**) Graphics displaying IL-12 (**E**), IL-6 (**F**), and IL-10 (**G**) concentrations in sera of patients with epilepsy in each cluster (*n* = 7–14, cluster 1; *n* = 26, cluster 2). (**H**) Distribution of inflammation assessed by CRP in patients with epilepsy in each cluster according to seizure frequency: No inflammation (CRP < 1, blue), slight inflammation (1 < CRP < 3, light red), significant (CRP > 3, red). (**I**) Histogram representing the percentage of immature neutrophils in cluster 1 (*n* = 10) and cluster 2 (*n* = 9) patients with epilepsy. (**J**) Radar chart illustrating the immune profiles of cluster 1 and cluster 2 based on IL-6, IL-12, immature neutrophils, and activation state of neutrophils. Each axis represents 1 immune parameter, and values correspond to the ratio of expression or frequency between cluster 1 and cluster 2, allowing visual comparison of immune characteristics across clusters. Normality was assessed using the Shapiro-Wilk test; comparisons were performed using 2-tailed Student’s t test or Mann-Whitney U test as appropriate. For distribution comparisons, the χ^2^ test was used. *P < 0.05, ***P < 0.001.

Regarding blood count, no significant differences in lymphocyte, neutrophil, or monocyte counts were found between the 2 clusters (Figure 6, A–D). Cytokine analysis revealed that cluster 1 had significantly higher levels of IL-6 and IL-10 compared with cluster 2 (IL-6: 1.3 vs. 0.7 pg/mL, *P* = 0.02; IL-10: 0.5 vs. 0.1 pg/mL, *P* = 0.03). Conversely, cluster 2 displayed significantly elevated levels of IL-12 (0.06 vs. 0.01 pg/mL, *P* = 0.02), a cytokine with well-established proinflammatory and immune-activating properties (Figure 6, E–G). Higher proportions of patients with DRE with CRP greater than 1 mg/mL or greater than 3 mg/mL were present in cluster 1 (χ^2^ = 43.01, *P* < 0.0001) (Figure 6H).

Given that IL-6 is a pleiotropic cytokine associated with emergency hematopoiesis (41), and that CXCR4^+^CD62L^+^ neutrophils are typically associated with steady-state or immature phenotypes (42), we further investigated the presence of immature neutrophils defined as CD15^+^CD10^–^. We found that patients in cluster 1 presented a significantly higher proportion of immature neutrophils in peripheral blood compared with cluster 2 (11.65 vs. 3.12 *P* = 0.02) (Figure 6I).

Taken together, these findings reveal the existence of 2 chronic low-grade inflammatory profiles among patients with DRE. Profile 1, associated with longer disease duration and lower seizure frequency, is characterized by a low-grade chronic inflammation enriched in immature neutrophils and elevated levels of pleiotropic (IL-6) and antiinflammatory (IL-10) cytokines. In contrast, profile 2, defined by higher seizure frequency, is marked by increased proportions of activated and hyperactivated neutrophils and elevated concentrations of the proinflammatory cytokine IL-12 (Figure 6J).

### Peripheral inflammation as a predictor of postsurgical epilepsy prognosis.

**Figure 7:**
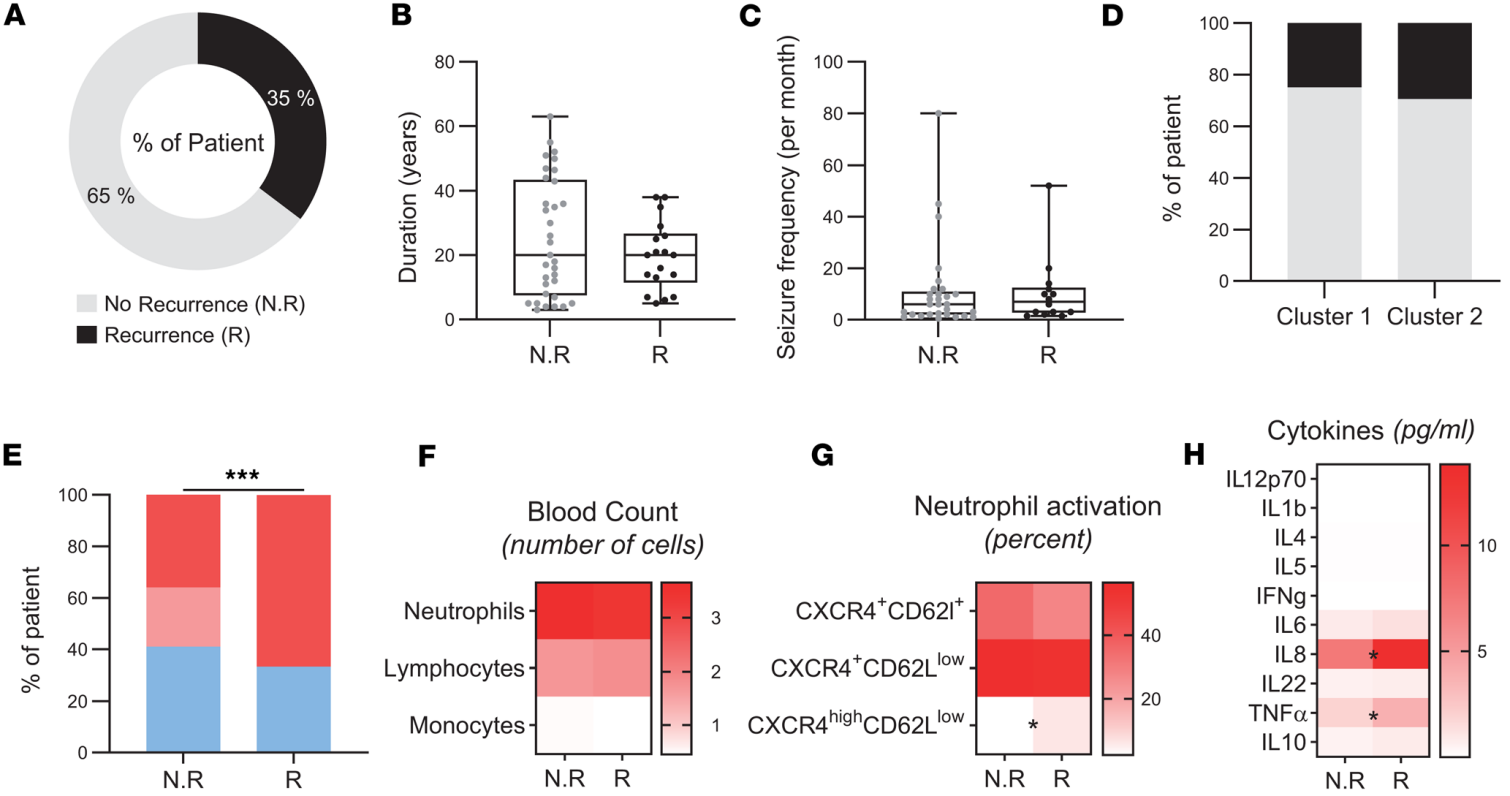
Peripheral inflammation as a predictor of postsurgical epilepsy prognosis. (**A**) Proportion of patients with recurrence (black) or non-recurrence (gray) of seizure within the year after surgery. (**B** and **C**) Graphics displaying clinical data disease duration (**B**), and seizure frequency per month (**C**) among patients with no seizure recurrence (N.R; gray, *n* = 33) or with seizure recurrence (R; black, *n* = 18). (**D**) Proportion of patients with recurrence (black) and non-recurrence (gray) of seizures among cluster 1 and cluster 2 patients with epilepsy. (**E**) Distribution of inflammation assessed by CRP in patients with epilepsy without (N.R) or with (R) recurrence: No inflammation (CRP < 1, blue), slight inflammation (1 < CRP < 3, light red), significant (CRP > 3, red). (**F**–**H**) Heatmap showing the leucocyte blood count (**F**), percentage of CXCR4^+^CD62L^+^, CXCR4^+^CD62L^lo^, or CXCR4^hi^CD62L^lo^ (**G**), and cytokine concentration (**H**) in blood of patients with epilepsy with or without seizure recurrence. Normality was assessed using the Shapiro-Wilk test; comparisons were performed using 2-tailed Student’s t test or Mann-Whitney U test as appropriate. For distribution comparisons, the χ^2^ test was used. ***P < 0.001.

Seizure recurrence after epilepsy surgery remains a major clinical challenge. In our cohort, 35% of patients experienced seizure recurrence within 1 year after surgery (Figure 7A). Several risk factors have been identified, such as seizure frequency, disease duration, and defined lesion (43). In our cohort, patients who presented with recurrent seizures did not exhibit a higher monthly seizure frequency or longer disease duration (Figure 7, B and C). Given the known role of inflammation in epileptogenesis, we hypothesized that specific immune and inflammatory parameters might serve as predictive biomarkers for seizure recurrence after surgery. We explored CRP levels as a marker of systemic inflammation. Patients who relapsed had significantly higher CRP values compared with seizure-free patients (χ^2^ = 30.02, *P* < 0,0001) (Figure 7E). However, the proportion of patients who experienced seizure recurrence 1 year after surgery was similar in cluster 1 and cluster 2 (Figure 7D).

**Figure 8:**
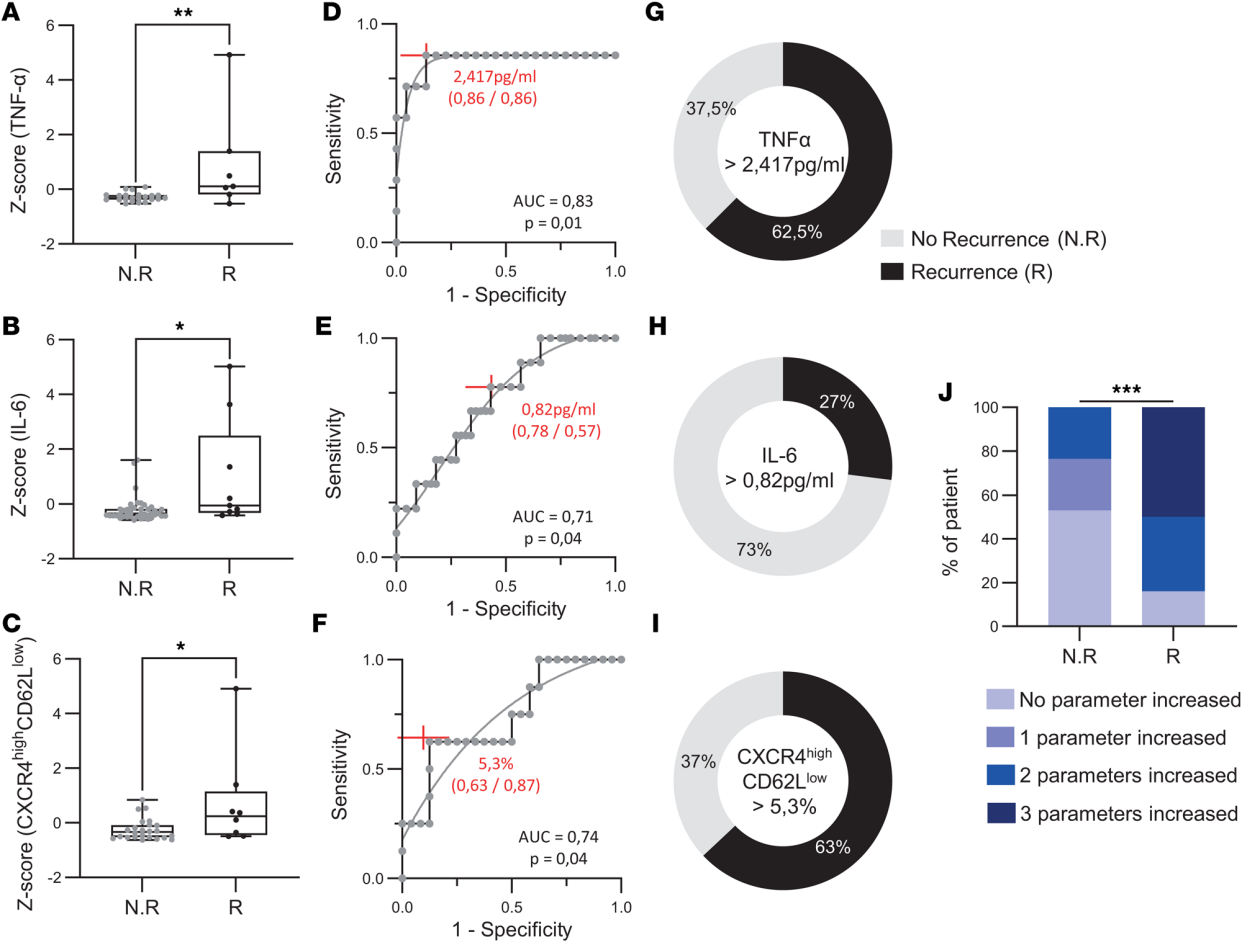
Peripheral inflammation as a predictor of postsurgical epilepsy prognosis. We calculated the *z* score for TNF-α, IL-6, and CXCR4^hi^CD62L^lo^ neutrophils (**A**–**C**) in blood from patients with DRE at preoperative evaluation comparing patients with (*n* = 8) and without (*n* = 24) postsurgery seizure recurrence at 1 year after surgery. (**D**–**F**) ROC curves illustrating the predictive performance of preoperative biomarkers for seizure recurrence 1 year after surgery. Each curve represents a distinct biomarker, and AUC reflects the overall discriminative ability. AUC values and corresponding *P* values are indicated for each marker. Youden index is plotted in red. (**G**–**I**) Proportion of patients with a TNF-α, IL-6, and percentage of CXCR4^hi^CD62L^lo^ neutrophils above the Youden index cutoff, stratified by seizure recurrence status 1 year after surgery. (**J**) Distribution of patients according to the number of elevated preoperative biomarkers (TNF-α, IL-6, and CXCR4^hi^CD62L^lo^ neutrophils) in relation to seizure recurrence 1 year after surgery. Normality was assessed using the Shapiro-Wilk test; comparisons were performed using 2-tailed Student’s t test or Mann-Whitney U test as appropriate. *P < 0.05, **P < 0.01, ***P < 0.001.

Analysis of complete blood count (CBC) revealed no significant differences in neutrophil, lymphocyte, and monocyte counts between the 2 groups (Figure 7F). However, analysis of serum cytokines showed that patients with postoperative seizure recurrence had significantly higher levels of IL-6 and TNF-α before the surgery (IL-6: 0.71 vs. 1.4; *P* = 0.02; TNF-α: 1.9 vs. 3.9; *P* = 0.007) (Figure 7H). Although the steady and activated phenotypes were comparable between groups, patients with seizure recurrence exhibited a significantly higher proportion of hyperactivated neutrophils (CXCR4^hi^CD62L^lo^) (6.4 vs. 2.2; *P* = 0.04) (Figure 7G). Normalization of *z* scores revealed a significant difference in preoperative IL-6 and TNF-α and CXCR4^hi^CD62L^lo^ levels in patients with seizure recurrence after surgery, supporting an association between the 3 inflammatory components and unfavorable postsurgical outcome (Figure 8, A–C).

Using ROC curve analysis, we assessed the predictive value of the 3 markers (i.e., TNF-α IL-6, CXCR4^hi^CD62L^lo^) for postoperative seizure recurrence. TNF-α showed the highest discriminative ability, with an AUC of 0.83 (95% CI, 0.5749–1.000; *P* = 0.01). IL-6 demonstrated moderate predictive accuracy, with an AUC of 0.71 (95% CI, 0.5472–0.8872; *P* = 0.04), and CXCR4^hi^CD62L^lo^ neutrophils showed a higher than IL-6 significant discriminative capacity, with an AUC of 0.74 (95% CI, 0.5388–0.9404; *P* = 0.04) (Figure 8, D–F). To further evaluate the clinical utility of each biomarker, the optimal cutoff values were determined using the Youden index. For TNF-α, the optimal threshold was 2.417 pg/mL, yielding a sensitivity of 0.86 and a specificity of 0.86 (Figure 8D). Among patients with concentrations above this cutoff, 63% experienced seizure recurrence within 1 year after surgery (Figure 8G). For IL-6, the optimal cutoff was 0.82 pg/mL (sensitivity 0.78, specificity 0.57), and 27% of patients above this threshold developed recurrent seizures (Figure 8, E–H). CXCR4^hi^CD62L^lo^ neutrophils had a lower sensitivity (0.63) but a higher specificity (0.87), and 63% of patients with values above the identified threshold experienced postoperative seizures (Figure 8, F–I). Each parameter was initially evaluated individually using the Youden index to determine optimal thresholds, and then these parameters were combined to improve the overall diagnostic accuracy. We evaluated the proportion of patients, with or without recurrence, who presented at least 1 of the 3 biomarkers above their respective predefined thresholds. This analysis was performed in the subgroup of patients with available values for all 3 parameters (*n* = 24). We found that 84% of patients with seizure recurrence had at least 1 elevated biomarker compared with 47% of patients without recurrence. Notably, 50% of patients with recurrence had all 3 biomarkers elevated, whereas none of the patients without recurrence showed this pattern (χ^2^ = 95.56, *P* < 0.0001) (Figure 8J). These findings suggest that the combined evaluation of these preoperative biomarkers may improve the identification of patients at higher risk of postoperative seizure recurrence.

## Discussion

To our knowledge, this is the first study to highlight a chronic low-grade systemic inflammation characterized by an altered activated neutrophil phenotype in DRE, with the identification of distinct immune profiles confirming the heterogeneity of peripheral immune responses in TLE. These profiles may serve as clinically accessible biomarkers for disease severity and postoperative outcomes.

Neuroinflammation has emerged as a key component in epilepsy pathophysiology. Although most studies have focused on central markers, peripheral blood offers a more accessible route for biomarker discovery. Our study highlights the presence of low-grade chronic systemic inflammation in DRE, confirming the elevated levels of TNF-α, IL-6, and CXCL8/IL-8 and neutrophil count, as previously described (26, 44). Monocytes and PBMCs are known contributors to systemic IL-12, IL-6, and TNF-α production (14, 45). Furthermore, when the BBB is dysfunctional, brain-derived cytokines may appear in the circulation, as suggested by correlations between IL-6 and TNF-α in cerebrospinal fluid and serum after tonic-clonic seizures (46, 47). The observed increase in neutrophil count in patients with epilepsy, particularly among those with recent seizures, could be attributed to the mobilization of marginated neutrophils and enhanced recruitment from the bone marrow. Elevated IL-6 levels may further contribute to this neutrophilia, as previously described in inflammatory conditions (48, 49).

We observed an increased proportion of CXCR4^+/hi^CD62L^lo^ neutrophils, positively associated with ROS production and negatively with neutrophil elastase, reflecting primary granule depletion in this highly activated, aged subset with enhanced migratory and inflammatory properties (50, 51). In our cohort, these cells were associated with high seizure frequency and longer disease duration, suggesting a potential role in chronic systemic inflammation in DRE, similar to their involvement in Alzheimer disease (21) and cerebral ischemia (20).

Importantly, this association appears to be independent of recent seizures, suggesting its potential value in reflecting overall disease severity in epilepsy. Although PCA suggested that neutrophil activation patterns were not primarily driven by seizure latency, these findings should be interpreted with caution. PCA is an exploratory approach and does not establish independence or causality. The limited contribution of latency may reflect cohort heterogeneity, limited sample size within etiological subgroups, or nonlinear temporal dynamics of inflammation that are not captured by this analysis. Larger studies focusing on more homogeneous patient populations (e.g., hippocampal sclerosis) and incorporating refined temporal modeling will be required to more rigorously assess the contribution of seizure latency and lesion type to peripheral inflammation and neutrophil activation. To minimize potential treatment-related confounding, patients receiving systemic glucocorticoids or immunotherapy were excluded, and no significant differences in inflammatory markers or neutrophil activation were observed according to antiseizure medication exposure, drug class, or polytherapy, suggesting that the observed immune profiles are not primarily driven by treatment effects. BMI data were not systematically collected for all participants; we acknowledge that differences in BMI could influence systemic inflammation and have included this as a limitation.

Chronic systemic inflammation has been identified as a factor increasing seizure susceptibility (4, 25) and contributing to the prevalence of epileptic seizures in chronic conditions such as lupus. It is noteworthy that reparixin, an inhibitor of CXCR1/CXCR2, the CXCL8/IL-8 receptors, has demonstrated reduced seizure frequency in rodent models of TLE (30). Moreover, previous studies have already suggested the relevance of inflammation in seizure propensity (52) and in the occurrence of seizures in posttraumatic epilepsy associated with elevated IL-1β (53). Chronic systemic inflammation has been associated with disease severity, and DRE with higher levels of IL-1β and IL-6 in monocytes in patients (14) or an increase of HMGB1 and TNF-α could be a risk of DRE (54).

In this study, we identified 2 systemic inflammatory profiles in patients with epilepsy. One was marked by elevated IL-12 and activated CXCR4^hi^CD62L^lo^ neutrophils, consistent with IL-12’s role as a neutrophil activator (55). In our cohort, IL-12 levels showed a positive, though nonsignificant, correlation with the proportion of activated neutrophils (Figure 1G). This profile was associated with higher seizure frequency, in line with clinical studies linking IL-12 to increased seizure burden (56) and elevated risk (57).

Other patients were defined by the presence of immature neutrophils (CD15^+^CD10^–^) along with elevated IL-6 levels, which appeared to correlate with a longer disease duration. This latter finding is consistent with previous reports of immature monocytes in patients with TLE, as opposed to those with chronic autoimmune limbic encephalitis (58), supporting the idea of a chronic inflammatory state evolving over time. Given the well-recognized plasticity of neutrophils and their ability to rapidly adapt to inflammatory cues (16), we cannot exclude that individual patients may transition between profiles over the course of the disease. However, the cross-sectional design of our study and the absence of longitudinal sampling preclude direct assessment of such intraindividual immune dynamics. These systemic inflammatory profiles not only deepen our understanding of the immune landscape in DRE, but also provide accessible, peripheral biomarkers that could support clinical stratification and prognosis. It should be noted that our gating strategy relied on forward scatter (FSC) and side scatter (SSC), along with CD15 expression, to define neutrophils, with confirmation of CD16 and CD66b expression. Although this approach is consistent with prior studies, the exclusion of these markers from the gating itself may limit the ability to fully capture activated or low-density neutrophil subsets. Future studies incorporating CD16 and CD66b into the gating strategy could improve neutrophil phenotyping accuracy, particularly for highly activated or atypical subsets (59–61), and provide further insight into their role in epilepsy-related inflammation.

Several studies have highlighted the risk of seizure recurrence after TLE surgery, particularly over the long term (62, 63). Contributing factors include incomplete resection of the epileptogenic zone, involvement of extratemporal or bilateral seizure networks, and the presence of focal cortical dysplasia (63). Additionally, clinical variables such as advanced age at the time of surgery, high preoperative seizure frequency, and earlier age at epilepsy onset have been identified as negative prognostic indicators (64, 65). However, these factors may not always be independently predictive and could instead reflect underlying structural pathology, such as hippocampal sclerosis (66). In line with this, we did not observe differences in postsurgical seizure recurrence between patients classified in cluster 1 or cluster 2, as these clusters primarily capture inflammatory profiles associated with seizure burden rather than surgical outcome. A key predictor of seizure recurrence remains the presence of postoperative EEG abnormalities, which markedly increase the risk of relapse (66).

Beyond neurophysiological markers, circulating molecular biomarkers are gaining attention. For example, specific miRNA signatures detected in blood samples have been associated with postoperative seizure outcomes (67). In this context, our findings suggest that elevated levels of TNF-α, IL-6, and a higher proportion of CXCR4^hi^CD62L^lo^ neutrophils may contribute to a higher probability of seizure recurrence, particularly when considered in combination rather than as isolated predictors. Supporting this concept, a recent study reported that postoperative neutrophilia after meningioma resection was associated with an increased risk of seizures (68), suggesting that systemic immune responses may modulate seizure outcomes. These findings collectively indicate that inflammatory markers in preoperative blood samples could serve as predictors of surgical outcomes in epilepsy.

It is acknowledged that patients may face challenges in accurately assessing their seizure frequency due to memory deficits (69) or an inability to perceive their seizures (70), which could be considered a limitation of the study. However, despite this limitation, we did not observe a correlation between recent seizures and neutrophil phenotype or cytokine levels. Our study characterizes the systemic immune response in patients with DRE, focusing on activated neutrophil phenotypes. These findings contribute to a growing body of evidence suggesting a role for innate immune mechanisms in epileptogenesis. By highlighting specific inflammatory pathways — particularly involving cytokines such as CXCL8/IL-8 and IL-6 — our results open promising avenues for further research into immunomodulatory treatment strategies. Such approaches may offer new therapeutic hope for patients with refractory epilepsy, for whom conventional treatments remain ineffective. Further studies are warranted to elucidate the role of chronic low-grade inflammation in epilepsy pathophysiology and to define distinct immune signatures that could serve as prognostic and/or diagnostic peripheral biomarkers.

## Methods

### Sex as a biological variable.

Both sexes (male and female) were included in the study. Given that no significant differences were observed between sexes, sex was not considered as a biological variable in the analyses.

### Experimental design.

The study was conducted in adult patients with DRE (*n* = 67, Table 1), who were under the care of the Epilepsy Unit at the Pitié-Salpêtrière Hospital, Paris, France. Blood samples were collected for 67 patients (Table 1). We defined baseline periods as times when samples were collected more than 48 hours after the last seizure (*n* = 31) and post-seizure periods as times when samples were collected within 48 hours after a seizure (*n* = 32).

Clinical data, such as demographics, medical history, treatments, seizure frequency, neutrophil and lymphocyte count, and CRP, were retrieved from medical records. The imaging and EEG findings were used to define the localization of the epilepsy, and the lesions were specified for the 49 patients who underwent surgery. Postsurgical recurrences were evaluated at 1 year after surgery for 48 patients. Patients receiving glucocorticoids or immunotherapy were excluded.

Age-matched healthy individuals, obtained from the établissement français du sang (EFS), were the controls (*n* = 35).

### Biospecimen collection.

For neutrophil phenotype analysis, blood samples were collected in lithium heparin tubes. Red-top blood tubes were used for cytokine analysis in serum and K2 EDTA tubes for blood cell count. Given that cytokine levels and neutrophil survival are affected by delayed analysis, all tubes were processed within 3 hours after collection (71). Whole blood analysis was preferred to avoid bias induced by cell isolation procedures (71).

### Neutrophil phenotyping.

The phenotypic analysis of neutrophils was performed using flow cytometry. Whole blood aliquots (100 μL) were incubated in the dark for 45 minutes at 4°C with different antibodies at the recommended concentration, according to the manufacturer’s instructions. RBCs were then lysed by adding 2 mL of lysis solution (BD Biosciences, 349202) to each tube for 5 minutes. After centrifugation (5 minutes at 440*g*, 300 μL of CellFix solution (BD Biosciences, 340181) was added. Samples were stored at 4°C pending flow cytometry analysis. Neutrophil phenotype was assessed using the following antibodies to 5 cell surfaces: CD62L-APC (BD Pharmingen, 559772, clone DREG-56), CXCR4-PECy7 (Sony Biotechnology, 2132570, clone 12G5), and CD10-PE (BD Pharmingen, 555375, clone HI10a). Viable cells were assessed with Fixable Viability Stain 620 (BD Horizon, 564996).

### Neutrophil elastase quantification.

Blood aliquots (1 mL) were centrifuged for 5 minutes at 440*g* to recover the serum for subsequent ELISA. The level of neutrophil elastase was quantified in the serum using a human neutrophil elastase ELISA kit (Abcam, ab270204) according to the manufacturer’s protocol.

### Analysis of ROS formation.

Oxidative explosion relates to the capacity of neutrophils to rapidly release reactive oxygen derivatives, such as superoxide ions and hydrogen peroxide. This process was assessed by flow cytometry using dihydroethidine (DHE) (Sigma-Aldrich, 37291), which is converted to fluorescence-emitting ethidium in the presence of oxygen. Blood aliquots (500 μL) were incubated for 15 minutes in a water bath at 37°C under agitation with DHE. RBCs were then lysed by adding 2 mL of lysis solution to each tube for 5 minutes. After centrifugation (5 minutes at 440*g*), 300 μL of CellFix solution was added to the tube and DHE fluorescence was analyzed by flow cytometry.

### Flow cytometry analysis.

All samples were analyzed using a Gallios flow cytometer (Beckman Coulter), and the acquired data were processed using Kaluza software (Beckman Coulter). To assess neutrophil phenotypes and functions, 50,000 events were acquired and analyzed within the neutrophil gate, identified based on FSC and SSC characteristics. Doublets were excluded based on FSC-H/FSC-A and SSC-H/SSC-A parameters, and viable cells were subsequently selected using a viability dye. Neutrophil identity was confirmed by the expression of CD15-BV510 (BD Biosciences, 567960, clone 7C3.rMAb), CD16-BV421 (BD Biosciences, 562874, clone 3G8), and CD66b Alexa Fluor 647 (BD Biosciences, 561645, clone G10F5) by this population. Within the viable cells, neutrophils were further classified into mature (CD10^+^) and immature (CD10^–^) subsets. Neutrophil activation status was determined based on the expression of CXCR4 and CD62L (Supplemental Figure 1).

### Cytokine measurement.

Red-top blood tubes were spun down at 1,500*g* for 10 minutes to harvest serum, which was subsequently stored at –80°C until further analysis. Serum cytokine concentrations were measured using the Human CorPlex Cytokine Panel Array kit on a Quanterix SP-X imaging and analysis platform (Sorbonne Université, Pitié-Salpêtrière Hospital).

### Statistics.

Data were analyzed using GraphPad Prism software. Before statistical analysis, outliers were evaluated using GraphPad Prism. Normality was assessed using the Shapiro-Wilk test. Based on the results, either Student’s 2-tailed *t* test or Mann-Whitney *U* test was performed. All statistical tests were bilateral with a type I error rate of 5%.

Correlation among cytokine/chemokine levels, demographics, and clinical data was evaluated by calculating Spearman’s *r* values and their level of significance.

PCA was performed as an exploratory multivariate analysis to identify patterns of association among clinical (age, disease duration, seizure frequency, and latency) and neutrophil activation (CXCR4^+^CD62L^+^, CXCR4^+^CD62L^lo^, CXCR4^hi^CD62L^lo^).

The distribution of patients across CRP-level subgroups was compared using a χ^2^ test. A *P* value less than 0.05 was considered statistically significant.

The prognostic performance of selected biomarkers was evaluated using ROC curve analyses. The AUC was calculated to assess the discriminative ability between patient subgroups (e.g., seizure recurrence vs. no recurrence). To determine the optimal threshold (cutoff) value for each marker, Youden’s index (J = sensitivity + specificity − 1) was calculated. The cutoff corresponding to the maximum Youden’s index was selected as the optimal point, maximizing both sensitivity and specificity. *z* scores were calculated for each biomarker using the mean and standard deviation of the full patient cohort. Standardized values were then compared between patients with and without postsurgical seizure recurrence.

Data are shown as box-and-whisker plots with individual values overlaid; boxes represent the IQR, the horizontal line indicates the median, and whiskers extend to the minimum and maximum values. Certain patients were not assessable for all parameters.

### Study approval.

All patients provided written consent. The study was sponsored by INSERM (C16-16, 2016-14388) and the protocol was approved by the Committee for the Protection of Persons in Ile de France.

### Data availability.

The data that support the findings of this study are available in the main text or the supplemental materials; values for all data points in graphs are reported in the Supporting Data Values file.

## Author contributions

CSDG, AH, VN, and MMB conceived and designed the study. CSDG, AH, KD, and G Gorochov acquired and analyzed data. AH, G Goudard, VM, VF, AB, and VN recruited patients. CSDG, GD, AH, VN, and MMB drafted a significant portion of the manuscript or figures.

## Conflict of interest

That authors have declared that no conflict of interest exists.

## Funding support

The following entities provided funding support.

- Agence nationale de la recherche (ANR) JCJC funding (ANR-21-CE14-0005-01) to MMB.
- “Investissements d’avenir” program (ANR-10-IAIHU-06) to VN.
- Fondation Assistance Publique-Hôpitaux de Paris (EPIRES–Marie Laure PLV Merchandising) to VN.

## Supplementary Material

Supplemental data

Supporting data values

## Figures and Tables

**Table 1:**
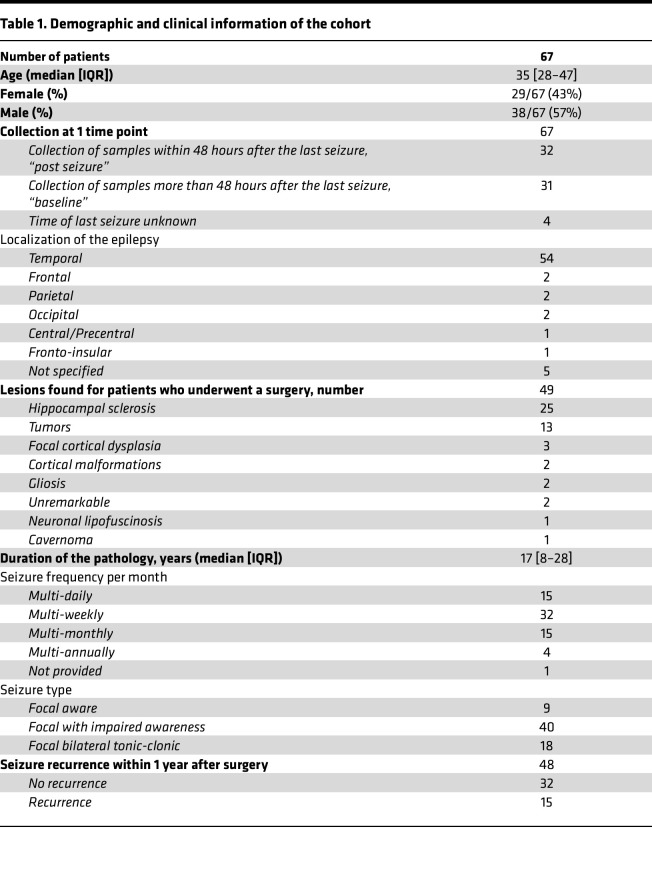
Demographic and clinical information of the cohort

**Table 2:**
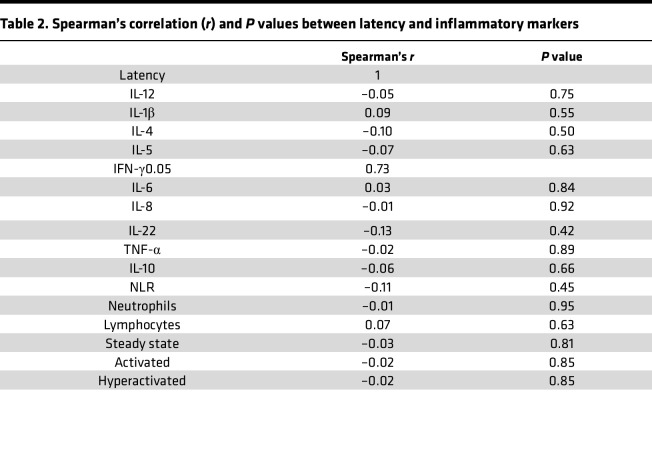
Spearman’s correlation (*r*) and *P* values between latency and inflammatory markers

## Version 1 04/14/2026

In-Press Preview

## Version 2 05/22/2026

Electronic publication
