# Supplementary material for: Systemic immune dysregulation and neutrophil activation define prognostic inflammatory signatures in drug-resistant epilepsy
Source: JCI Insight. 2026 Apr 14;11(10):e200419. doi: 10.1172/jci.insight.200419 (PMC13232733; doi:10.1172/jci.insight.200419)
Supplement: Supplemental data [file jciinsight-11-200419-s164.pdf]

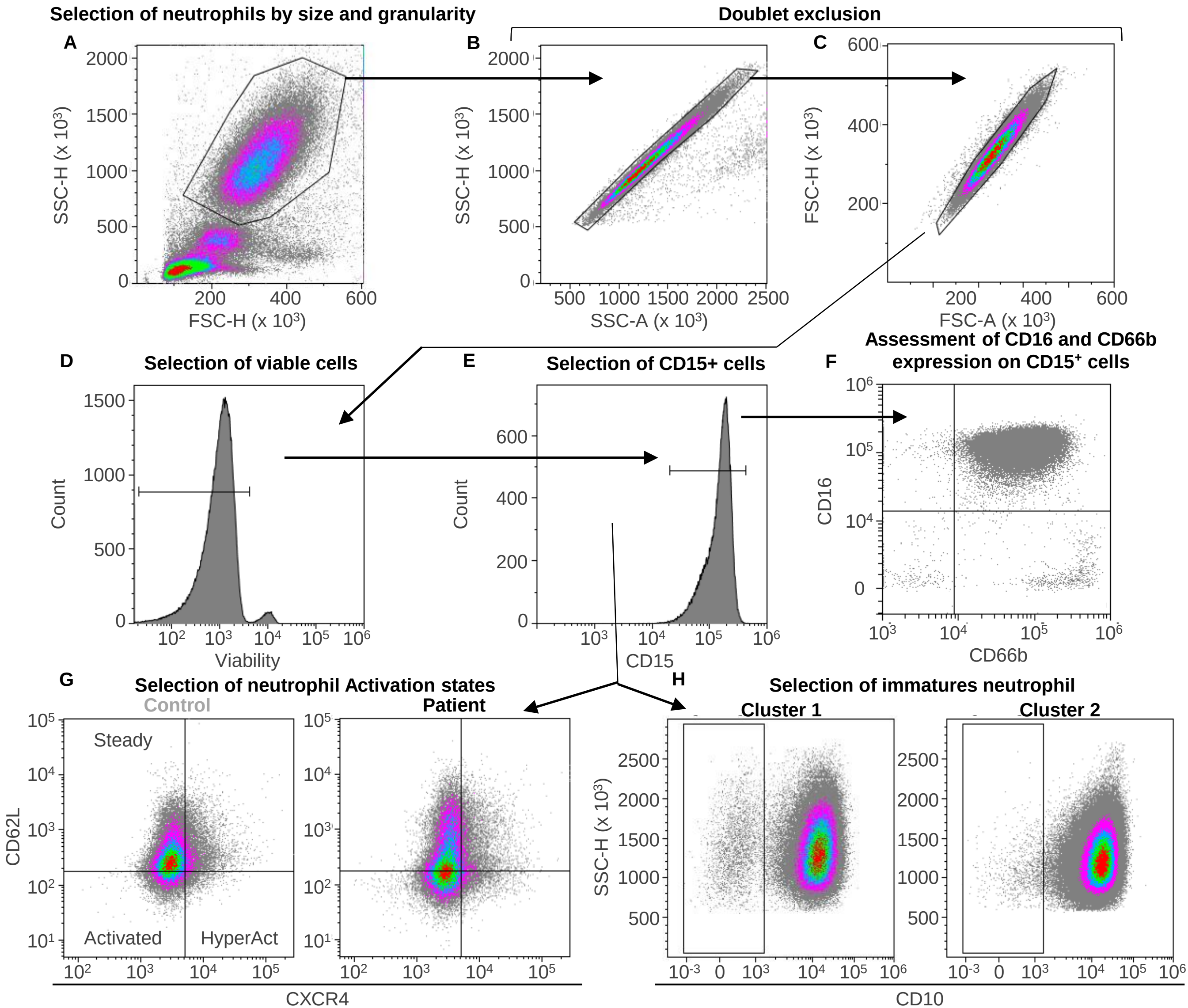

Supporting Figure 1: Gating strategy for flow cytometry analysis of neutrophils (A) Initial identification of the neutrophil population based on forward scatter (FSC) and side scatter (SSC) properties.**(B–C)** Doublet exclusion within the neutrophil gate using SSC-H/SSC-A followed by FSC-H/FSC-A parameters.**(D)** Selection of viable cells.**(E)** Selection of $CD15^+$ cells. **(F)** Assessment of CD16 and CD66b expression on $CD15^+$ cells. **(G)** Identification of neutrophil activation states: steady ($CXCR4^+CD62L^+$), activated ($CXCR4^+CD62L^{low}$), and hyperactivated ($CXCR4^{high}CD62L^{low}$).**(H)** Detection of immature neutrophils based on $CD10^-$ expression.

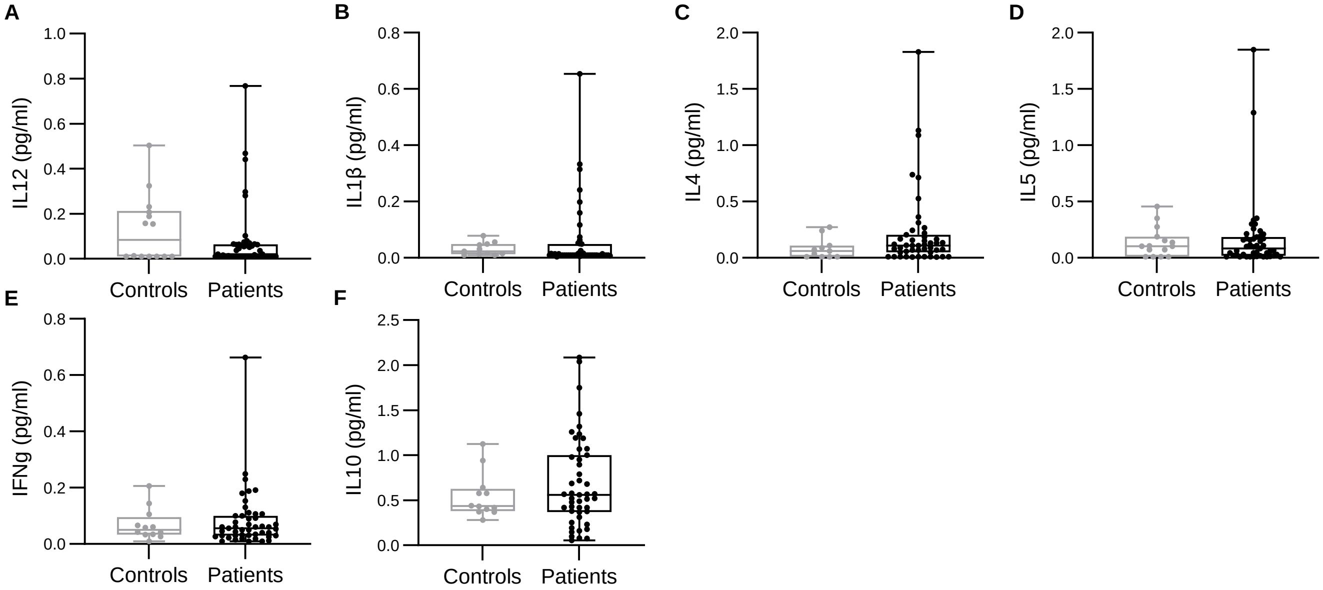

Supporting Figure 2 : Cytokines concentration in sera of patients with epilepsy

(A-F) Graphics displaying cytokines concentrations in sera of control (grey) and epileptic patients (black) and expressed in pg/ml. Data are shown as box-and-whisker plots with individual values overlaid; boxes represent the interquartile range, the horizontal line indicates the median, and whiskers extend to the minimum and maximum values.

Data are shown as box-and-whisker plots with individual values overlaid; boxes represent the interquartile range, the horizontal line indicates the median, and whiskers extend to the minimum and maximum values. Normality was assessed using the Shapiro-Wilk test; subsequently, Student’s t-test or Mann-Whitney test was conducted.*P<0.05, **P<0.01, ***P<0.001.

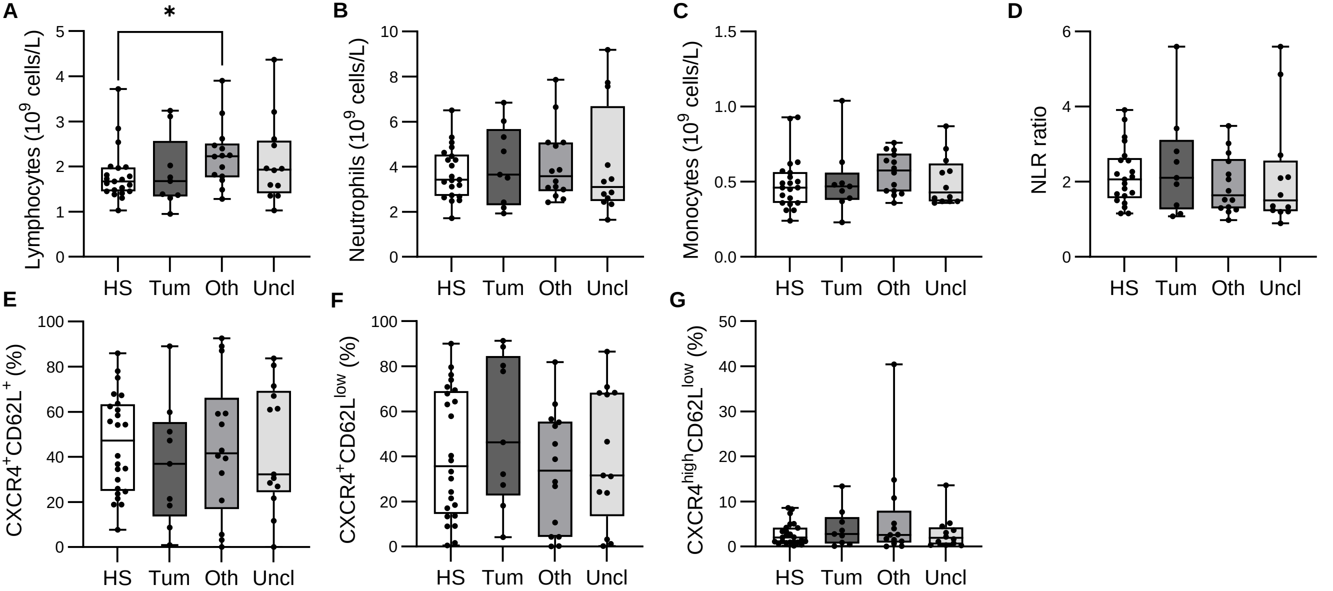

Supporting figure 3 : Neutrophil activation state depending on localization and type of lesion

(A-D) Quantification of lymphocytes **(A)**, neutrophils **(B)**, monocytes **(C)**, and neutrophil-to-lymphocyte ratio **(D)** in whole blood from patients with epilepsy with Hippocampal sclerosis (HS), **(E-G)** Percent of CXCR4$^+$CD62$^+$ **(E)**, CXCR4$^+$CD62Llow **(F)** and CXCR4^{high}CD62L^{low} **(G)** neutrophils in patients with epilepsy with Hippocampal sclerosis (HS) or others lesion including tumors. Data are shown as box-and-whisker plots with individual values overlaid; boxes represent the interquartile range, the horizontal line indicates the median, and whiskers extend to the minimum and maximum values. Normality was assessed using the ShapiroWilk test; subsequently, Student's t-test or Mann-Whitney test was conducted. *P<0.05, **P<0.01, ***P<0.001.

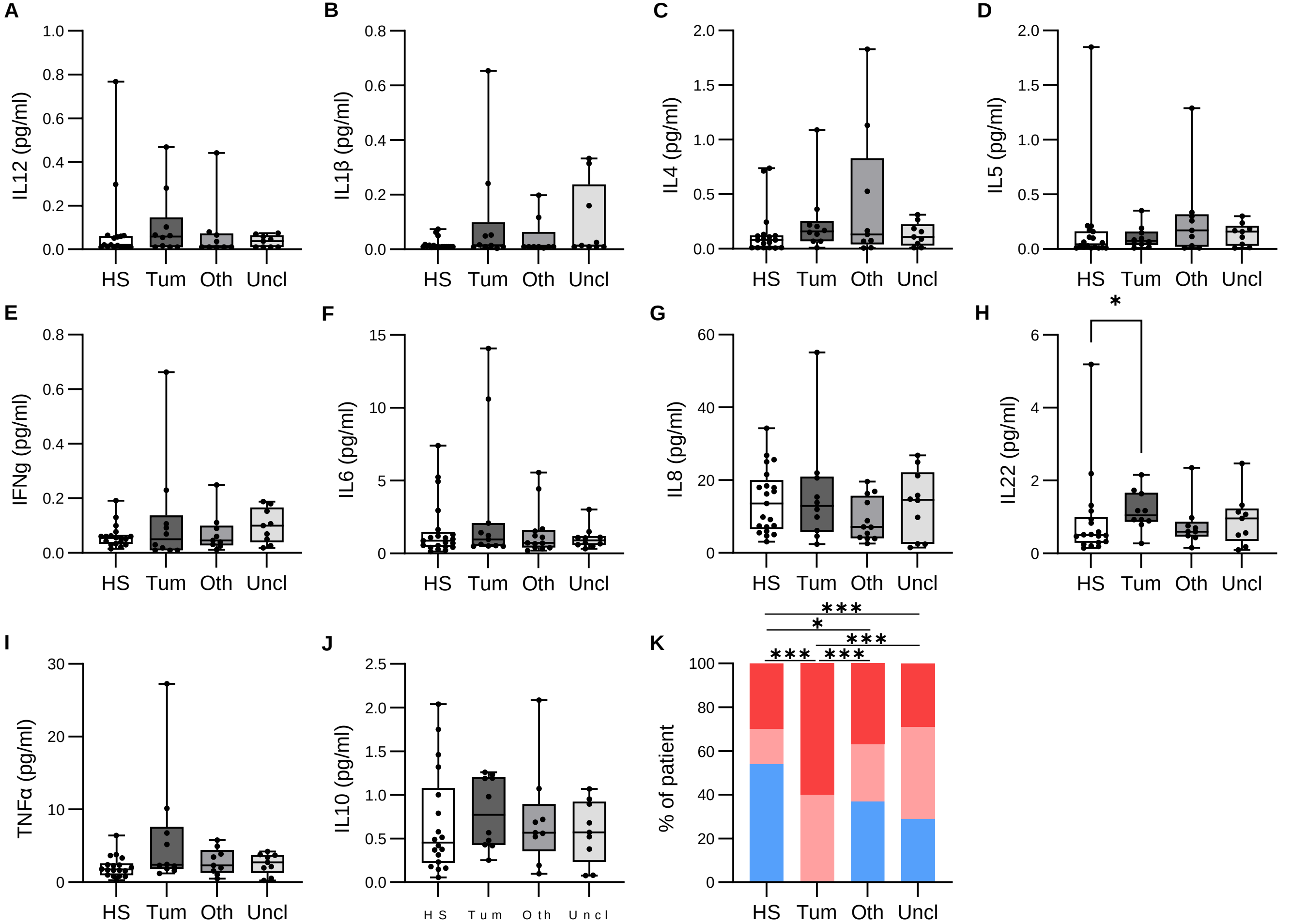

Supporting figure 4 : Chronic peripheral low-grade inflammation independent localization and type of lesion

(A-J) Graphics displaying cytokines concentrations in sera of epileptic patients with Hippocampal sclerosis (HS), Tumors (Tum) others lesion (Oth) or unclassified lesion (Uncl) and expressed in pg/ml. **(K)** Distribution of inflammation assessed by CRP in epileptic patients with Hippocampal sclerosis (HS) or others lesion including tumors. No inflammation (CRP<1, blue), Inflammation: slight (1<CRP<3, light red), significant (CRP>3, red). Data are shown as box-and-whisker plots with individual values overlaid; boxes represent the interquartile range, the horizontal line indicates the median, and whiskers extend to the minimum and maximum values. Normality was assessed using the ShapiroWilk test; subsequently, Student's t-test or Mann-Whitney test was conducted. For distribution comparisons, the Chi-squared test was used. *P<0.05, **P<0.01, ***P<0.001.
